# Supplementary material for: Farming for Pharming: Novel Hydroponic Process in Contained Environment for Efficient Pharma-Grade Production of Saffron
Source: Molecules. 2022 Dec 16;27(24):8972. doi: 10.3390/molecules27248972 (PMC9784412; doi:10.3390/molecules27248972)
Supplement: Supplementary file 1 [file molecules-27-08972-s001.zip › molecules-1986524-supplementary.pdf]

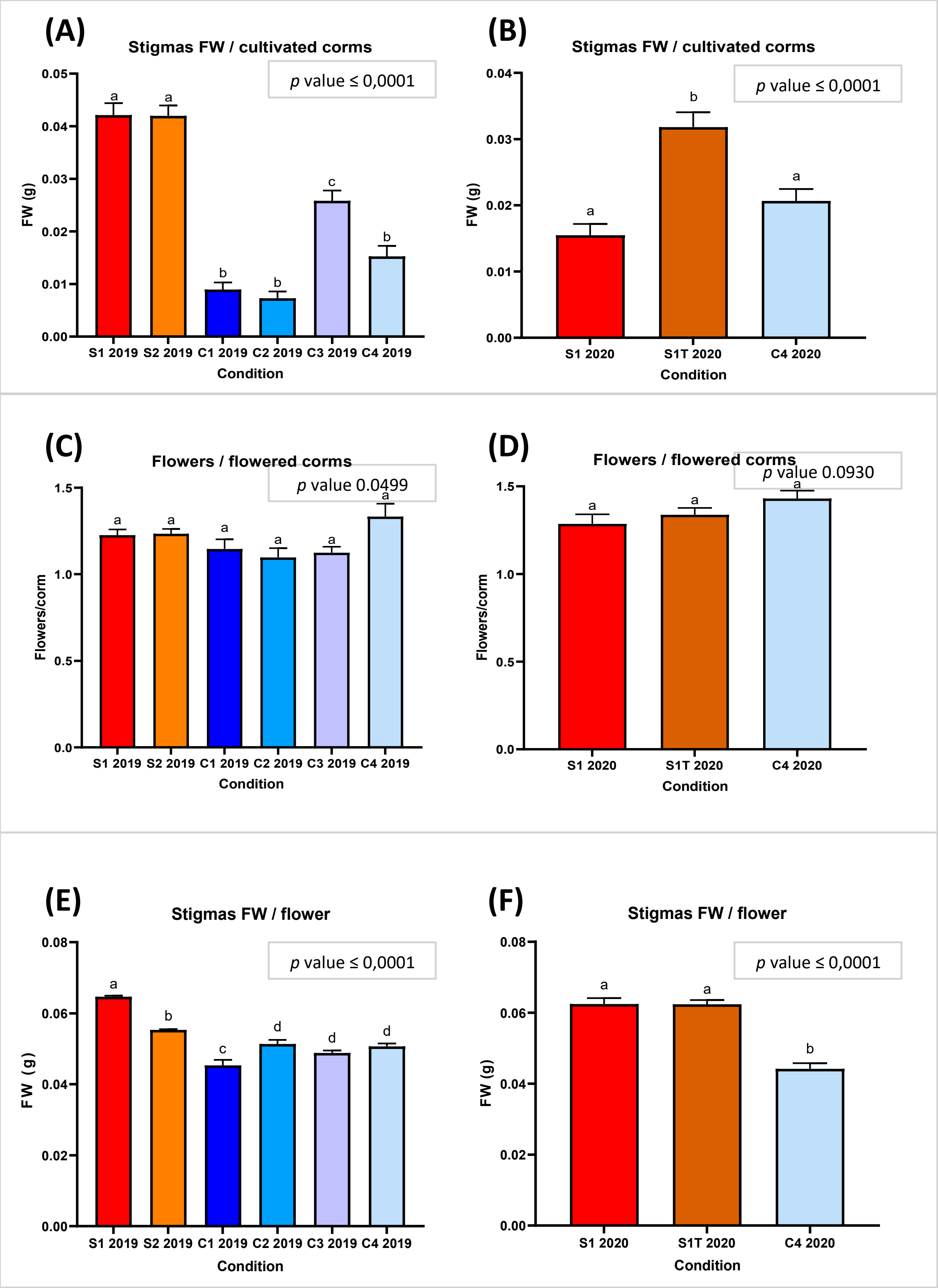

Figure S1. Statistical analysis results that complement the data shown in Figure 3. Mean ratio between the fresh weight (FW) of the collected stigmas and the cultivated corms, in the first (2019) **(A)** and in the second (2020) **(B)** experimental cycle. Mean of the ratio between flowering obtained and flowered corms, in the first (2019) **(C)** and in the second (2020) **(D)** experimental cycle. Mean of the fresh weight (FW) of the collected stigmas per flower, in the first (2019) **(E)** and in the second (2020) **(F)** experimental cycle. Standard error of the mean (SEM) was calculated by the Tukey-test and statistical significance in indicated in the grey box. Identical lowercase letters, reported at the top of each bar, correspond to means of experimental conditions that gave results without statistically significant differences.
